# Supplementary material for: Potential Role of Phytochromes A and B and Cryptochrome 1 in the Adaptation of Solanum lycopersicum to UV-B Radiation
Source: Int J Mol Sci. 2023 Aug 24;24(17):13142. doi: 10.3390/ijms241713142 (PMC10488226; doi:10.3390/ijms241713142)
Supplement: Supplementary file 1 [file ijms-24-13142-s001.zip › ijms-2547426-supplementary.pdf]

Table S1. Table of primers used in the research.

Gene Bank ID	Gene description	Gene	Primer 5′-3′	
			Forward	Reverse
NM_001320396.1	SPA nuclear gene for chloroplast product	*SPA*	AGAACACTCAGCCCTGCTTC	GGTACCGAGGTTGAATGCCA
EU218537.1	Cullin 4	*CUL*	AGCCAGAACAAAGCTCCCAA	ACGCCTACCACTGTACTTGC
XM_004238269.4	B-box protein 21	*BBX21*	GCAGCTTCATGTAGTGCGAA	ACAGAACCAGAAACAGGGGA
A0A3Q7E952	phytochromobilin synthase	*HY2*	ATGAAAGCTCTGGTGTTTCTTA C	CCGTCGGTCCCTTCAATACAT
Solyc04g074180	cryptochrome 1	*CRY1*	TCCTGAACTTGCTAGGCTGC	CAGCTGCATCATTCTGCCAC
Solyc09g090100	cryptochrome 2	*CRY2*	TTGGGGACCTGGAAAACGAG	TTCCCCCGACTCTCAACCTA
NM_001374394.1	anthocyanidin synthase	*ANT*	TCTCAATTCCCACCTCGCAC	ACTTTGCGCTCAGCAAGAAC
Solyc06g060340.3.1	S subunit PSII	*PSBS*	TGTTGGCCGTGTTGCTATGA	TTGCCAGGAGGGATAACAGC
AH001371.2	chlorophyll a/b binding protein 1	*CAB1*	CTGAAGACCCGGAGGCATTT	TGCAAGGTGATCAGCAAGGT
YP_008563068.1	photosystem II protein D1	*psbA*	TGAAGGTTACAGATTCGGTCA	TGAATATGCAACAGCAATCCA
YP_008563083.1	photosystem II protein D2	*psbD*	GATATTATGGATGACTGGTTA CG	CATTCTCTGGTCCCATTGC
NM_001306202.1	phytochrome B1	*PHYB1*	GCTCAATCTTCAGGCGTGG	CCCAGTAACATCCTGCCCAA
XM_019214123.2	ultraviolet - B receptor	*UVR8*	TTCGACGGTTGACCCATCAG	TCCAGTTCCTCTTTCGGGAAT
XM_015201575.2	repressor of UV-B photomorphogenesis 2	*RUP2*	ATCGGTATGGAAACCGGGTG	ATGTCTTGGTTCGCGTCCAT
Solyc09g075080.3.1	Phytochrome a-associated f-box protein	*PHYA-F*	CCGTGAGCAGGGAAATAAGC	GATCGATCTTCCTATTGCCATCA
NM_00124747191.2	Short hypocotyls 5	*HY5*	ACCCGACCCAAACATTGTCG	GGCTAGGGTTAATGGCGGTT
NM_001247104.2	Chalcone synthase	*CHS*	CCGTGGACCCAGTGAATCTC	AGAGTTTGGGCTGCTGAGAC
XM_004249510.4	Phenylalanine ammonia-lyase 1	*PAL1*	GAAGCGTTCCATGTTGCTGG	TCAGTGAACTCGGGCTTTCC
NM_001247883.2	Phytoene synthase	*PSY*	CGGGGAATTTGGGCTTGTTG	CCACCTATCTAAGGCTGCCG
NM_001308008.1	Phytochrome-interacting factor 4	*PIF4*	AATCTGGAACTGCCCGAAGG	GCCACTTCCCATCCACATCA
A0A3Q7F8W6	Tubulin alpha chain	*TUB*	ACAACTTTGCCCGTGGACAT	TGCTCAAGAAGGGAGTGGGT
NM_001247118.2	E3 ubiquitin-protein ligase	*COP1*	ATAATTTGCCGGTTCGCACG	CACGGGTTTGGATATCGCCT
NM_001247219.2	De-etiolated1	*DET1*	TGATGGCTTCCTTGCCTTGT	CCATCTGTGCTGCCAGCTTC
